# FBXO11 and FBXO32 synergistically activate innate immunity against hepatitis B virus in a NEDD8-dependent manner

**DOI:** 10.3389/fimmu.2026.1833429

**Published:** 2026-09-03

**Authors:** Liqiang Li, Shun Tao, Yunfei Zhang, Zihan Zeng, Liang Li, Jun Zhang

**Affiliations:** 1 Department of General Surgery, The Second People’s Hospital of Hefei, Hefei Hospital Affiliated to Anhui Medical University, Hefei, China; 2 Department of General Surgery, Hefei Second People’s Hospital Affiliated to Bengbu Medical University, Bengbu, China

**Keywords:** FBXO11, FBXO32, HBV, innate immunity, NEDD8, synergistic effect, TRAF3, ubiquitination

## Abstract

**Background and objective:**

Type I interferon (IFN-I) signaling is crucial for antiviral innate immunity. F-box protein-mediated ubiquitination regulates this pathway. FBXO11 positively regulates IFN-I signaling via TRAF3 K63-linked ubiquitination, but whether other F-box proteins act synergistically during HBV infection remains unknown. This study investigates FBXO32 expression and function in HBV infection, and whether FBXO11 and FBXO32 synergistically activate innate immunity through a NEDD8-dependent mechanism.

**Methods:**

Immunofluorescence (IF) staining for HBcAg and HBsAg confirmed successful HBV infection. qRT-PCR screening of 14 FBXO family members in HBV-infected HepG2-NTCP cells identified FBXO11 and FBXO32 as the most markedly upregulated. Cellular loss- and gain-of-function studies (single or double knockout and overexpression) were performed, followed by qRT−PCR and western blot analysis of antiviral genes. Mechanistically, co-immunoprecipitation was used to assess FBXO11/FBXO32 interactions with TRAF3, TBK1, and IRF3, and the NEDD8 inhibitor MLN4924 was applied to confirm NEDD8 dependence. *In vivo*, to distinguish progeny viral DNA from the injected plasmid, a control experiment was performed in which the vector backbone sequence of the pAAV/HBV1.2 plasmid was detected alongside the HBV gene. Subsequently, hydrodynamic tail-vein injection of the pAAV/HBV1.2 plasmid was performed in C57BL/6 mice with single or double knockout or overexpression of FBXO11 and FBXO32.Serum and liver samples were collected at 0, 6, 12, 24, and 48 hours post-infection to quantify HBV DNA (qPCR), HBsAg, HBeAg and HBcAg (ELISA). Liver pathology was evaluated by H&E staining, and immunohistochemistry (IHC) for HBcAg, ISG56 and CXCL10 was performed on liver tissue sections.

**Results:**

HBV infection markedly upregulated FBXO11 and FBXO32. Double knockout suppressed antiviral genes more strongly than single knockout. Mechanistically, FBXO11 and FBXO32 cooperatively enhanced NEDD8-dependent K63-linked ubiquitination of TRAF3, promoting TRAF3-TBK1-IRF3 complex assembly. Control experiments confirmed that the detected HBV DNA predominantly represented progeny virus rather than input plasmid. Based on this validated system, double knockout of FBXO11 and FBXO32 exacerbated liver pathology and elevated viral antigens, whereas double overexpression alleviated pathology and reduced antigens.

**Conclusion:**

FBXO11 and FBXO32 synergistically enhance antiviral innate immunity by promoting NEDD8-dependent K63-linked TRAF3 ubiquitination, suppressing HBV replication and alleviating immunopathology. These findings reveal a synergistic anti-HBV axis and provide a potential therapeutic strategy.

## 1 Introduction

Hepatitis B virus (HBV) infection remains a major global health challenge and is a leading cause of chronic hepatitis, cirrhosis, and hepatocellular carcinoma (1–3). Recent epidemiological data indicate that despite universal vaccination programs, millions of people are still living with chronic HBV infection worldwide, underscoring the urgent need for a deeper understanding of host antiviral mechanisms (1, 2). The host’s innate immune response, particularly the rapid and robust activation of the type I interferon (IFN-I) system, is critical for the early control of HBV infection and viral clearance (4, 5). Emerging evidence has highlighted that the magnitude and kinetics of IFN-I production directly influence the outcome of HBV infection, from spontaneous resolution to chronic persistence (4, 6).

IFN-I production is tightly regulated through a multi-layered signaling cascade. Pattern recognition receptors (PRRs) are the key sensors of the innate immune system that recognize pathogen-associated molecular patterns (PAMPs) from invading viruses and bacteria (7, 8). Among the various PRR families, Toll-like receptors (TLRs) represent a well-characterized and crucial class of membrane-bound sensors that bridge innate and adaptive immunity, and they have been implicated in the recognition of HBV components (9, 10). This core pathway is initiated by PRRs such as RIG-I and cGAS (11, 12), transduced via mitochondrial antiviral signaling protein (MAVS) or stimulator of interferon genes (STING) (13, 14), and culminates in the phosphorylation of interferon regulatory factor 3 (IRF3) by TANK-binding kinase 1 (TBK1), which is essential for antiviral defense (15, 16). Tumor necrosis factor receptor-associated factor 3 (TRAF3) acts as a pivotal adaptor protein in this cascade (17, 18). Notably, TRAF3 is a central and essential component of TLR signaling, particularly in the pathways that lead to type I interferon production (19, 20). In the TLR3-TRIF signaling axis, TRAF3 is recruited to the signaling complex and facilitates the activation of TBK1 and subsequent phosphorylation of IRF3 (21, 22). TRAF3 also participates in TLR7/9-mediated signaling through the MyD88-dependent pathway, further underscoring its indispensable role in bridging TLR activation to IFN-I induction (22, 23). The post-translational modification status of TRAF3, especially ubiquitination, directly modulates the efficiency of TBK1–IRF3 axis activation (24, 25).

Ubiquitination is a crucial post-translational modification that extensively participates in the initiation, amplification, and feedback regulation of innate immune signaling (26, 27). The SCF (SKP1-Cullin-F-box) complex is a multi-subunit E3 ubiquitin ligase whose substrate specificity is conferred by its F-box protein component (28, 29). Recent studies have revealed that several F-box proteins are involved in the regulation of antiviral immunity (30, 31). Specifically, FBXO11 was recently identified as a positive regulator of IFN-β production by promoting K63-linked polyubiquitination of TRAF3, thereby facilitating TRAF3–TBK1–IRF3 complex assembly and downstream signaling—a process dependent on the ubiquitin-like protein NEDD8 (27). This finding established FBXO11 as a novel positive modulator of innate immunity. Nevertheless, the F-box protein family comprises numerous members with diverse functions (32, 33), and whether and how other members participate in antiviral immunity—particularly whether functional synergy or antagonism exists in specific pathological contexts such as HBV infection—remains largely unexplored.

FBXO32, also known as Atrogin-1 or MAFbx, is another important F-box protein best recognized for its role in muscle atrophy, where it mediates the ubiquitin-proteasome-dependent degradation of specific substrates such as MyoD and eIF3-f (34, 35). Recent work has also implicated FBXO32 in inflammatory and stress responses in immune cells (36, 37), yet its specific function in antiviral innate immunity, particularly within the IFN-I signaling pathway, has not been elucidated. Intriguingly, our preliminary experiments and expression screening indicated that HBV infection concurrently upregulates the expression of both FBXO11 and FBXO32, implying that these two F-box proteins may coordinately respond to viral infection and play interrelated roles in immune regulation.

Based on these observations, we hypothesize that during HBV infection, FBXO11 and FBXO32 act synergistically to co-regulate the TRAF3-centered innate immune signaling node, thereby amplifying the host’s antiviral defense. To test this hypothesis, we first systematically profiled the expression of FBXO family members following HBV infection at the cellular level and validated the synergistic effect of FBXO11 and FBXO32 on antiviral gene expression. We then investigated whether they operate through a shared molecular mechanism—namely, the NEDD8-dependent regulation of TRAF3 ubiquitination. Finally, using an *in vivo* mouse model, we comprehensively evaluated the synergistic protective effects of FBXO11 and FBXO32 co-expression against HBV infection from pathological and molecular perspectives.

## 2 Method

### 2.1 Cell culture and knockout

HepG2-NTCP and HepG2.2.15 cells were all purchased from Xiamen Yimo Biotechnology Co., Ltd. Both cell lines were maintained in DMEM supplemented with 10% fetal bovine serum at 37 °C in a 5% CO_2_ atmosphere. To knock out FBXO11,FBXO32 in HepG2-NTCP cells, we used the CRISPR/Cas9 system.

A short guide RNA (sgRNA) targeting FBXO11,FBXO32 was designed and cloned into a Lenti-V2 plasmid. The sgRNA sequence were (FBXO11)5’-AAACTTACATCTGCTCCCTCCC-3’,(FBXO32)5’-AAATATGCTCATCCTCCCTCCC-3.

### 2.2 Preparation of HBVcc

Culture supernatant of HepG2.2.15 cells was collected and concentrated using a 10,000 MWCO Millipore Ultra-15 centrifugal filter unit at 4 °C and 5,000 rpm. The concentrated virus (HBVcc, genotype D) was aliquoted and stored at -80 °C. Prior to use, HBV DNA was extracted from the culture supernatant using a viral nucleic acid extraction kit and quantified by PCR. The working concentration was adjusted to 1×10^6^ copies/mL.

### 2.3 HBV infection *in vitro*

HepG2-NTCP cells in the logarithmic growth phase were harvested, centrifuged at 1,000 rpm for 5 min, and resuspended in complete medium. After counting, cells were seeded into collagen-coated 6-well plates at 2×10^5^ cells per well and incubated at 37 °C. Following attachment, the medium was replaced with Williams’E medium containing 10% FBS, 1% penicillin/streptomycin, 2% DMSO, and 1% glutamine. After 24 h, the medium was renewed. Frozen HBVcc was thawed on ice, and cells at 30-50% confluence were infected at a multiplicity of infection (MOI) of 80. If necessary, the virus stock was diluted with PBS to ensure accurate pipetting. The infection medium (2 mL per well) consisted of Williams’ E medium supplemented with 10% FBS, 1% penicillin/streptomycin, 2% DMSO, 1% glutamine, and 4% PEG8000, plus the calculated virus volume. After discarding the original medium, the infection medium was added to each well, mixed gently, and incubated for 24 h. The cell supernatant was then collected.

### 2.4 Western blot analysis

The following primary antibodies were used for immunoblotting: anti-FBXO11 (Abcam, ab181801) at 1:500, anti-FBXO32 (Bioss, bs-2591R) at 1:1000, anti-TRAF3 (Bioss, bs-1185R) at 1:1000, anti-IRF-3 (CST, 4302S) at 1:1000, anti-TBK1 (CST, 3504S) at 1:1000, anti-p-TBK1 (Bioss, bs-3440R) at 1:1000, anti-p-IRF3 (Proteintech, 80519-2-RR) at 1:2000, anti-IFNB1 (Bioss, bs-23732R) at 1:1000, anti-IFNA4 (Bioss, bs-42189R) at 1:1000, anti-ISG56 (Proteintech, 83423-1-RR) at 1:10000, anti-CXCL10 (Proteintech, 85307-2-RR) at 1:5000, anti-IL-6 (Wanleibio, WL02841) at 1:1000, and anti-GAPDH (Zsbio, TA-08) at 1:5000. All antibodies were diluted according to the manufacturers’ instructions or as indicated. Protein lysates were prepared from clinical samples, mouse liver tissues, or transfected HepG2-NTCP cells using RIPA buffer. Equal amounts of protein were separated by SDS-PAGE and transferred to PVDF membranes. After blocking, membranes were incubated overnight at 4 °C with primary antibodies against FBXO11, FBXO32, TRAF3, phospho-TBK1, total TBK1, phospho-IRF3, total IRF3, IFNB1, IFNA4, ISG56, CXCL10, IL-6, and GAPDH (loading control). Following incubation with HRP-conjugated secondary antibodies, protein bands were visualized by ECL and quantified using ImageJ software.

### 2.5 qRT-PCR analysis

Total RNA was extracted using TRIzol reagent and reverse transcribed into cDNA. Gene expression was analyzed by quantitative PCR with SYBR Green chemistry on a real-time PCR system. Relative mRNA levels were calculated using the 2^(-ΔΔCt) method, normalized to GAPDH. The primer sequences are listed in **Table 1**. The extraction of viral DNA from serum was performed using the EasyPure Viral DNA/RNA Kit (TransGen Biotech).

**Table 1:** Primer sequences used for qRT-PCR analysis.

Gene	Forward primer(5’→3’)	Reverse primer(5’→3’)
β-actin	CCCTGGAGAAGAGCTACGAG	GGAAGGAAGGCTGGAAGAGT
FBXO2	AGCGACCCTGCCTCCACTCT	AAGCAAACCTATGCCAACAAA
FBXO4	AAAATCCAGCCGTCCTAT	GTTGGGCAAAGTTCCTCT
FBXO5	CAGTGGCTATTCCTCATT	CTGGGCTTTGTATTTGTA
FBXO6	GTCCGCTACATCCTCTTCC	CCTGGTTCCTGGTCATCTT
FBXO11	AGGTCCTGGTGCACAAAATA	GGTGCTGCTGATAGATCTTG
FBXO17	GACTGGACTACTGCCTGACG	CACCATGCCTGGCTAATT
FBXO22	CGACCTCAGGAAATAGAA	ACCAAAGACAAGGACCAC
FBXO24	CCCCTGAGACCTAATCCC	CCCTGAACATTCTTACCCT
FBXO27	AAACCCACAAGTGAGCAT	TGAAGGGACCAAGGAAGT
FBXO30	CTTTGTAATGGCTTTCCT	TTTCCCTAAGTTGTGCTG
FBXO32	GTCTCCCCATCCGTCTAGT	GCTCTTCTCATCCAGGAAGC
FBXO34	TCGGTAAAGCATCATCTC	AGTGGTCCTTCTTCTTCG
FBXO38	ATCCAGCCTTCCCTAACC	TACCACATCAGCCAAACC
FBXO45	TCCCATAGCTGTTAGAGTC	AATAGATTTGCCTTCCTTC
Ifnb1	CCTACAAAGAAGCAGCAA	CCTCAGGGATGTCAAAGT
Ifna4	CCAGTTCCAGAAGGCTCA	TTCCAGGTCATTCAGTTGC
Isg56	GCTCACGCCTGTAATCTC	CTCCAGCAGTCCACTCACC
Cxcl10	GTCAAGCCATAATTGTTC	ATAGTGCCAGGGTAGAGT
Il6	GGGCTCTTCGGCAAATGTAG	TGCCCAGTGGACAGGTTTCT
TRAF3	CCGCGAGAACTCCTCTTTC	ACTTGTACTTGTCCTCCACG
TBK1	GGAAGTGTCCTGAGTCTCG	CCACGAAAGACATTTGCAGT
IRF3	ACAGCAGGAGGATTTCGG	ATGTGGGTCGTGAGGGTC
HBV	CACCTCTGCCTAATCATC	GGAAAGAAGTCAGAAGGCAA
GAPDH	GCACCGTCAAGGCTGAGAAC	TGGTGAAGACGCCAGTGGA

### 2.6 Co-immunoprecipitation assays

Protein lysates were immunoprecipitated overnight at 4 °C using specific antibodies against FBXO11, FBXO32, TRAF3, TBK1, or IRF3, followed by incubation with Protein A/G beads. Normal IgG served as negative control. After washing, bound proteins were eluted and analyzed by Western blotting to detect interacting partners. Reciprocal Co-IP was performed to validate specific interactions between FBXO11/FBX032 and components of the TRAF3-TBK1-IRF3 pathway.

### 2.7 Establishment of HBV-infected mouse model and grouping

All mice were housed under SPF conditions, and all procedures were approved by the Animal Experimental Ethics Committee of Anhui Medical University (LLSC20232168). A total of 160 male C57BL/6 mice (6–8 weeks old) were used and divided into two batches. The first batch consisted of normal control(n=15) and HBV infection (n=15)groups. These mice were used to establish the time course of HBV infection. Mice in the HBV infection group were injected via the tail vein with 20 μg of pAAV/HBV1.2 plasmid dissolved in normal saline at a volume equivalent to 10% of the mouse’s body weight, while the normal control group received empty vector. The entire injection process was completed within 5–8 seconds (38). Blood samples were collected from the retro-orbital sinus at 0 h (pre-injection), 6 h, 12 h, 24 h, and 48 h post-injection, and liver tissue was harvested at the same time points. HBV DNA was extracted from both serum and liver tissue and quantified by PCR to confirm successful model establishment. The second batch was used for the gene knockout and overexpression experiments. CRISPR/Cas9-FBXO11, CRISPR/Cas9-FBXO32, CRISPR/Cas9-FBXO11/FBXO32double knockout,pCMV-Myc-FBXO11,pCMV-Myc-FBXO32,pCMV-Myc-FBXO32/FBXO11 double overexpression mice were purchased from GemPharmatech Co., Ltd. Mice were randomly assigned to fourteen experimental groups (n=8 per group): normal control, HBV infection, CRISPR/Cas9-FBXO11,HBV+CRISPR/Cas9-FBXO11,CRISPR/Cas9-FBXO32,HBV+CRISPR/Cas9-FBXO32,CRISPR/Cas9-FBXO32+CRISPR/Cas9-FBXO11,HBV+CRISPR/Cas9-FBXO32+CRISPR/Cas9-FBXO11,pCMV-Myc-FBXO11, HBV+pCMV-Myc-FBXO11,pCMV-Myc-FBXO32,HBV+pCMV-Myc-FBXO32,pCMV-Myc-FBXO32+pCMV-Myc-FBXO11,andHBV+pCMV-Myc-FBXO32+pCMV-Myc-FBXO11. All mice in the second batch were injected with pAAV/HBV1.2 plasmid (or empty vector for controls) following the same protocol, and all samples were collected at 24 h post-infection.

### 2.8 Histopathological analysis

Formalin-fixed tissues were embedded in paraffin, sectioned at 4-μm thickness, and mounted on glass slides. Sections were deparaffinized, rehydrated, and stained with hematoxylin and eosin (H&E) according to standard protocols. Stained sections were examined under a light microscope (Olympus IX71) to evaluate inflammatory cell infiltration, tissue edema, degeneration, and other pathological changes induced by HBV infection.

### 2.9 Immunohistochemistry

Paraffin-embedded tissue sections were deparaffinized and subjected to antigen retrieval using citrate buffer (pH 6.0). Endogenous peroxidase activity was blocked with 3% H_2_O_2_. Sections were incubated overnight at 4 °C with primary antibodies against FBXO11, FBX032, Isg56, Cxcl10, HBcAg. After washing, sections were incubated with HRP-conjugated secondary antibodies, developed with diaminobenzidine (DAB), and counterstained with hematoxylin.

### 2.10 Elisa assays

Levels of HBsAg(ml106737, 2025081,mlbio,Shanghai),HBeAg (ml002205, 20250810,mlbio,Shanghai) and HBcAg (JL49885,Jianglai,Shanghai)in in serum were quantified using commercial ELISA kits according to the manufacturers’ protocols. All assays were performed in duplicate, with absorbance measured at 450 nm using a microplate reader. Antigen concentrations were calculated based on the standard curves provided with each kit.

### 2.11 Immunofluorescence

HepG2-NTCP cells were fixed on coverslips with 4% paraformaldehyde for 30 min, permeabilized with 0.3% Triton X-100 for 20 min, and blocked with goat serum for 30 min. Cells were incubated with primary antibodies against HBcAg (bs-15455R,Bioss) at 37 °C for 60 min, followed by HRP-conjugated secondary antibodies and TSA fluorescence dye reaction (10 min). Nuclei were counterstained with DAPI for 5 min. Coverslips were mounted with anti-fade mounting medium and images were captured using a fluorescence microscope.

### 2.12 Statistical analysis

Data are presented as mean ± standard error of the mean (SEM). Statistical comparisons between two groups were performed using Student’s t-test viaGraphPad prism 9.0 (San Diego, California, USA), with P<0.05 considered statistically significant.

## 3 Result

### 3.1 FBXO11 and FBXO32 synergistically exert antiviral effects

**Figure 1:**
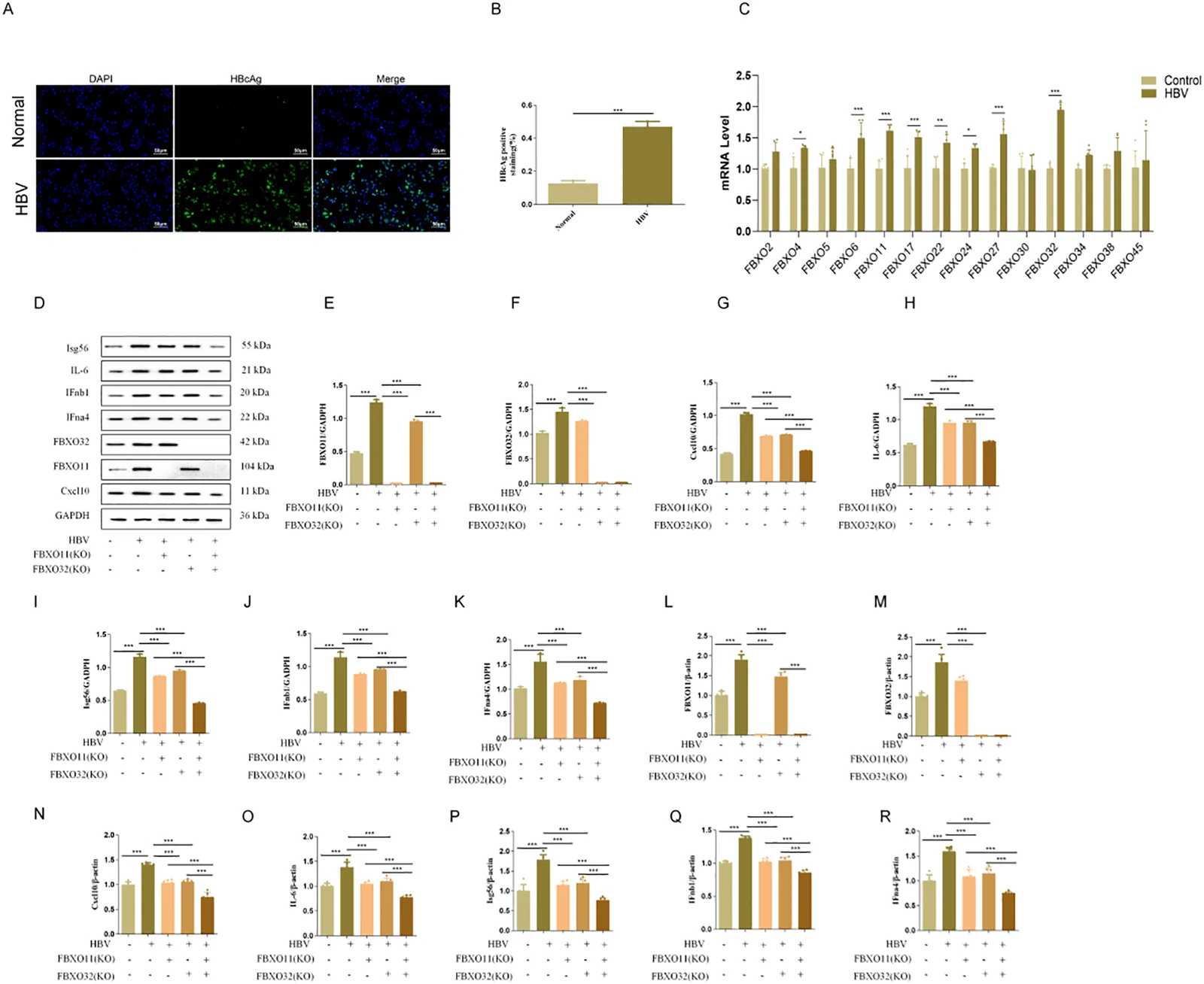
FBXO11 and FBXO32 synergistically exert antiviral effects. **(A, B)** The levels of HBcAg were measured by IF in HepG2-NTCP cells. **(C)** The mRNA levels of FBXO2, FBXO4, FBXO5, FBXO6, FBXO11, FBXO17, FBXO22, FBXO24, FBXO27, FBXO30, FBXO32, FBXO34, FBXO38, FBXO45 were measured by PCR in HepG2-NTCP cells. **(D–K)** The protein levels of FBXO11, FBXO32, Cxcl10, Il6, Isg56, Ifnb1, Ifna4 were measured by Western blot in HepG2-NTCP cells. **(L–R)** The mRNA levels of FBXO11, FBXO32, Cxcl10, Il6, Isg56, Ifnb1, Ifna4 were measured by PCR in HepG2-NTCP cells. n ≥3, *P<0.05, **P<0.001, ***P<0.0001.

To systematically investigate the expression of the FBXO family following HBV infection, we infected HepG2-NTCP cells with HBV. Immunofluorescence assays first confirmed that the expression of HBcAg was enhanced upon HBV infection (**Figures 1A, B**). The results further showed HBV infection induced the upregulation of multiple FBXO members, with the most pronounced increase observed in the mRNA transcript levels of FBXO11 and FBXO32 (**Figure 1C**). Given their marked upregulation, we next sought to determine whether FBXO11 and FBXO32 function cooperatively in antiviral immunity. Further loss-of-function experiments revealed that, compared with individual knockout, double FBXO11 and FBXO32 significantly suppressed the expression of antiviral genes, including Ifnb1, Ifna4, Isg56, Cxcl10, and Il6 (**Figures 1D–R**). These findings indicate that, following HBV infection, FBXO11 and FBXO32 synergistically and positively regulate the innate immune signaling pathway, thereby enhancing the host antiviral response.

### 3.2 FBXO11 and FBXO32 facilitate antiviral innate immunity by enhancing TRAF3 ubiquitination in a NEDD8-dependent manner

**Figure 2:**
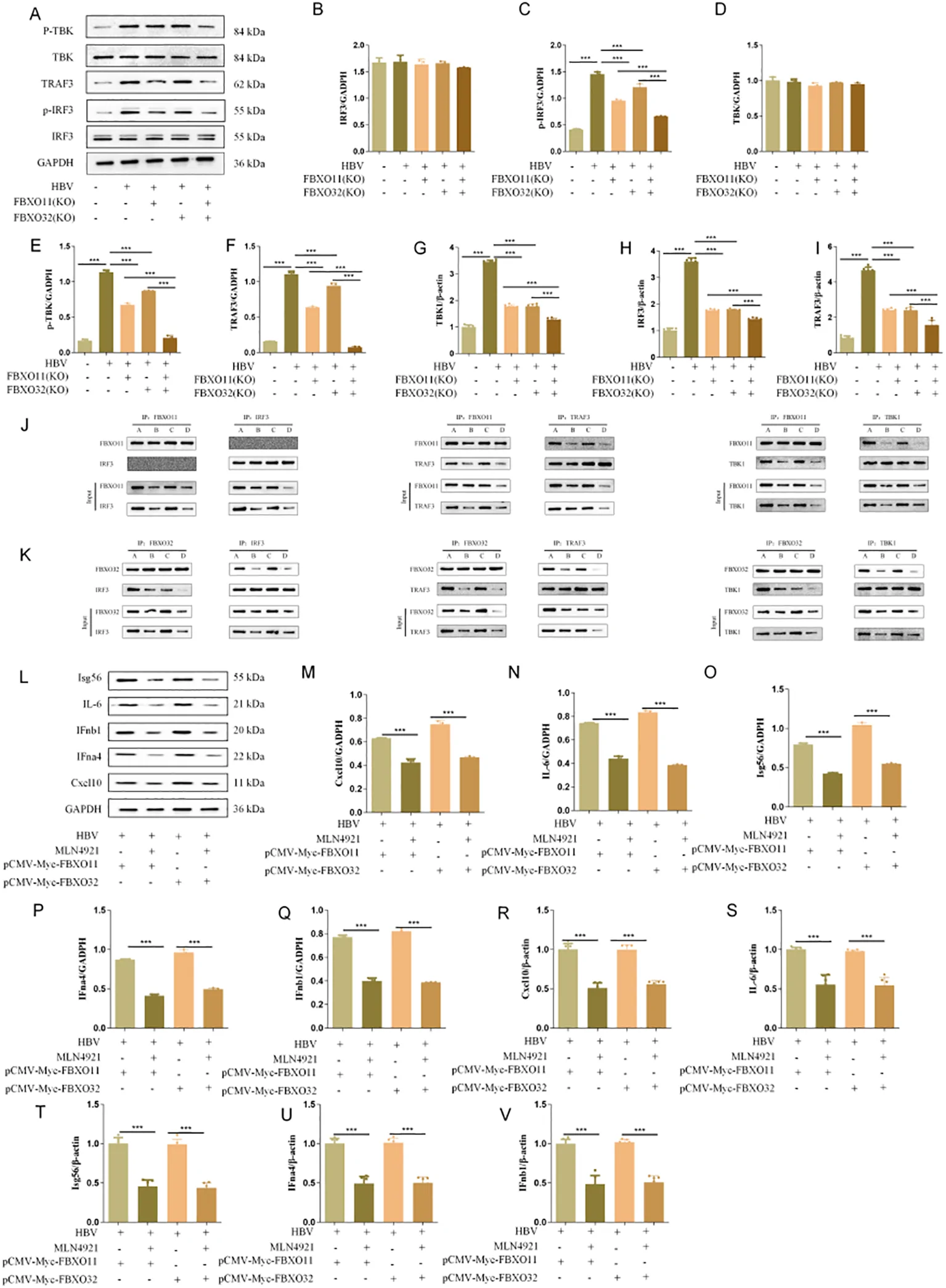
FBXO11 and FBXO32 facilitate antiviral innate immunity by enhancing TRAF3 ubiquitination in a NEDD8-Dependent manner. **(A–F)** The protein levels of IRF3, p-IRF3, TBK1, p-TBK1, TRAF3 were measured by Western blot in HepG2-NTCP cells. **(G–I)** The mRNA levels of TBK1, IRF3, TRAF3 were measured by PCR in HepG2-NTCP cells. **(J)** Co-IP of FBXO11 and TRAF3, FBXO11 and TBK1, FBXO11 and TBK1 in HepG2-NTCP cells. (A group: HBV+pCMV-Myc-FBXO11, B group: HBV+pCMV-Myc-FBXO11+MLN4924, Cgroup: HBV+pCMV-Myc-FBXO32,D group: HBV+pCMV-Myc-FBXO32+MLN4924). **(K)** Co-IP of FBXO32 and, TRAF3 FBXO32 and TBK1, FBXO32 and TBK1 in HepG2-NTCPcells. **(L–Q)** The protein levels of FBXO11, FBXO32, Cxcl10, Il6, Isg56, Ifnb1, Ifna4 were measured by Western blot in HepG2-NTCP cells. **(R–V)** The mRNA levels of FBXO11, FBXO32, Cxcl10, Il6, Isg56, Ifnb1, Ifna4 were measured by PCR in HepG2-NTCPcells. n ≥3, ***P<0.0001.

Subsequently, we aimed to investigate whether FBXO11 and FBXO32 synergistically regulate innate immune activation through a similar NEDD8-dependent mechanism. We first examined the expression of key components of the TRAF3–TBK1–IRF3 axis in cells with single or double knockout of FBXO11 and FBXO32. The results showed that the protein levels of TRAF3, TBK1, and IRF3 were significantly lower in double−knockout cells than in single−knockout cells (**Figures 2A–I**). Next, cells overexpressing both FBXO11 and FBXO32 were treated with MLN4924, a specific inhibitor of NEDD8. MLN4924 suppressed the assembly of the TRAF3–TBK1–IRF3 complex mediated by FBXO11 and FBXO32 (**Figures 2J, K**), accompanied by reduced expression of downstream antiviral genes(Cxcl10, Il6, Isg56, Ifnb1, Ifna4) (**Figures 2L–V**). These findings further corroborate the critical role of NEDD8 in the signaling cascade regulated by FBXO11 and FBXO32.

### 3.3 Double knockout of FBXO11 and FBXO32 during HBV infection impairs synergistic innate immunity, leading to elevated viral antigen levels and exacerbated liver pathology

**Figure 3:**
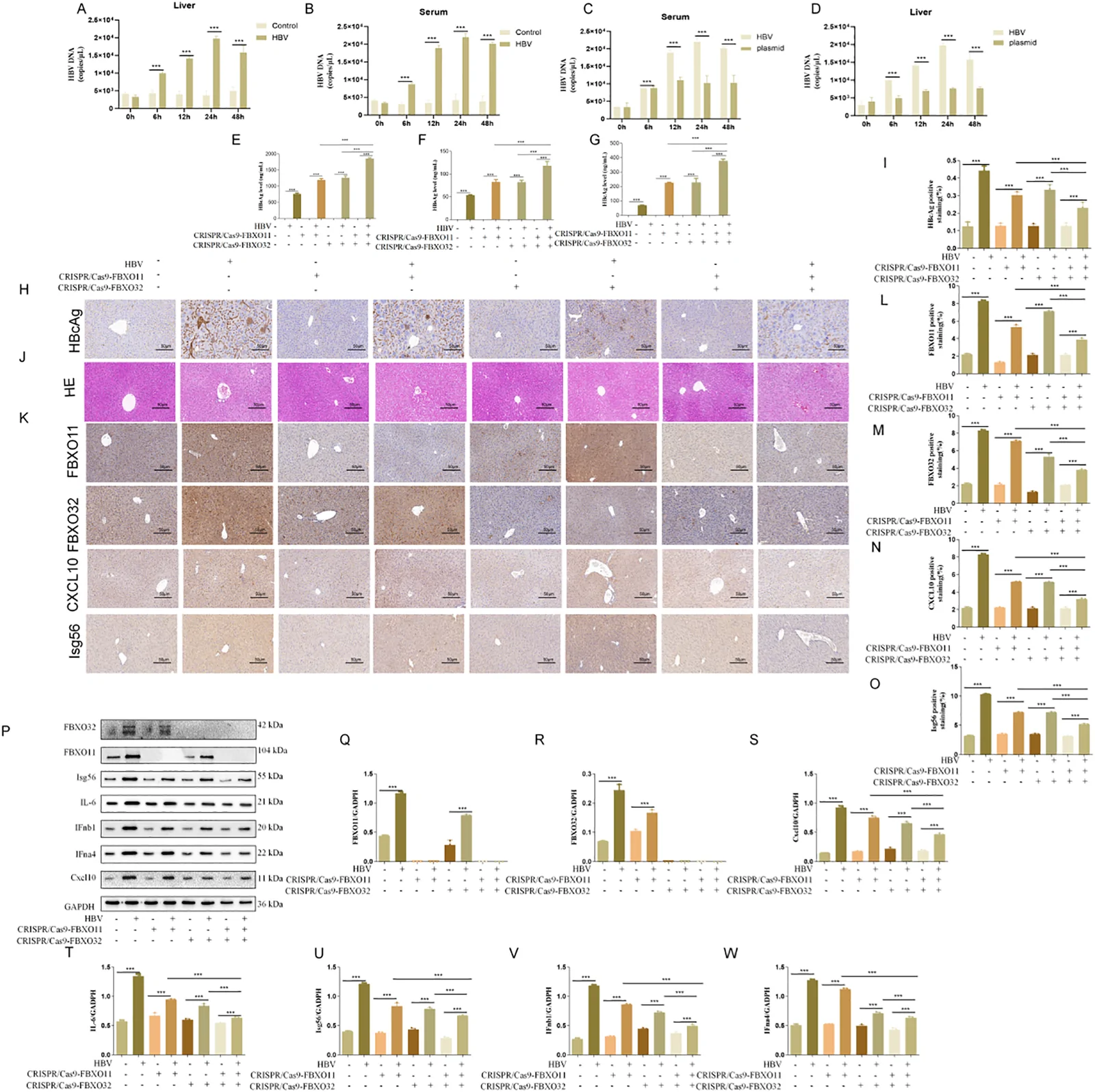
Double knockout of FBXO11 and FBXO32 during HBV infection impairs synergistic innate immunity, leading to elevated viral antigen levels and exacerbated liver pathology. **(A)** The level of HBV DNA in liver tissues from mice at 0h,6h,12h,24h,48h post-HBV infection. **(B)** The level of HBV DNA in mice serum at 0h,6h,12h,24h,48h post-HBV infection. **(C)** The level of HBV DNA in liver tissues in HBV group and plasmid from mice at 0h,6h,12h,24h,48h post-HBV infection. **(D)** The level of HBV DNA in mice serum at 0h,6h,12h,24h,48h post-HBV infection. **(E–G)** The level of HBsAg,HBeAg and HBcAg in mice serum at 24h post-HBV infection. **(H, I)** The level of HBcAg in liver tissues from mice at24h post-HBV infection. **(J)** HE staining of liver tissues from mice at 24 post-HBV infection. **(K–O)** Immunohistochemical staining results of FBXO11, FBXO32, and immune-related markers (Isg56, Cxcl10) in liver tissues from mice at 24h post-HBV infection. **(P–W)** The protein levels of FBXO11, FBXO32, Cxcl10, Il6, Isg56, Ifnb1, Ifna4 were measured by Western blot in mice at 24h post-HBV infection. n ≥3, ***P<0.0001.

To further corroborate the synergistic function of FBXO11 and FBXO32 in anti-HBV immunity, we performed loss-of-function experiments by knocking out FBXO11 and/or FBXO32 *in vivo*. We first measured HBV DNA levels in both serum and liver tissue at various time points post−infection. Compared with the uninfected control group, HBV DNA was detectable as early as 6 hours post-infection, peaked at 24 hours, confirming successful viral replication and expression *in vivo*, and indicating that the model was properly established (**Figures 3A, B**). To distinguish progeny viral DNA from the injected plasmid, we performed a control experiment in which, in addition to standard HBV DNA detection, we also detected the vector backbone sequence of the same pAAV/HBV1.2 plasmid used for model injection. As shown in **Figures 3C, D**, in the liver, the vector backbone signal did not increase over time; in the serum, it showed a modest increase at 6 hours and plateaued after 12 hours. In contrast, the standard HBV DNA signal continued to rise and remained elevated at 24 hours. These results indicate that the majority of HBV DNA detected at the 24-hour time point represents progeny viral DNA from active replication rather than residual input plasmid. Therefore, the 24-hour time point was selected for subsequent experiments. Upon HBV infection, serum HBsAg, HBeAg and HBcAg levels were markedly elevated; however, single knockout of either FBXO11 or FBXO32 further increased both antigen levels. Notably, double knockout led to even higher antigen levels, achieving a greater increase than single-gene knockout (**Figures 3E–G**). Immunohistochemical staining for HBcAg in liver tissue was consistent with the serum findings (**Figures 3H, I**). Accordingly, histopathological analysis revealed that HBV-induced inflammatory infiltration, edema, and degeneration were partially aggravated by single knockout and substantially worsened by double knockout (**Figure 3J**). Consistent with these pathological changes, immunohistochemical staining showed that the antiviral effectors ISG56 and CXCL10, which were moderately induced by HBV alone, were reduced by single knockout and decreased to even lower levels upon double knockout (**Figures 3K–O**). At the molecular level, Western blot analysis confirmed that the upregulation of Ifnb1, Ifna4, ISG56, CXCL10, and IL-6 was significantly impaired by single knockout, with an even more pronounced reduction observed after double knockout (**Figures 3P–W**). In the absence of HBV infection, no significant changes in any measured parameters were observed regardless of knockout. Collectively, these loss-of-function experiments demonstrate that FBXO11 and FBXO32 act synergistically to dampen antiviral immunity, promote HBV replication, and exacerbate virus−induced liver pathology.

### 3.4 Double overexpression of FBXO11 and FBXO32 during HBV infection enhances synergistic innate immunity, leading to reduced viral antigen levels and alleviated liver pathology

**Figure 4:**
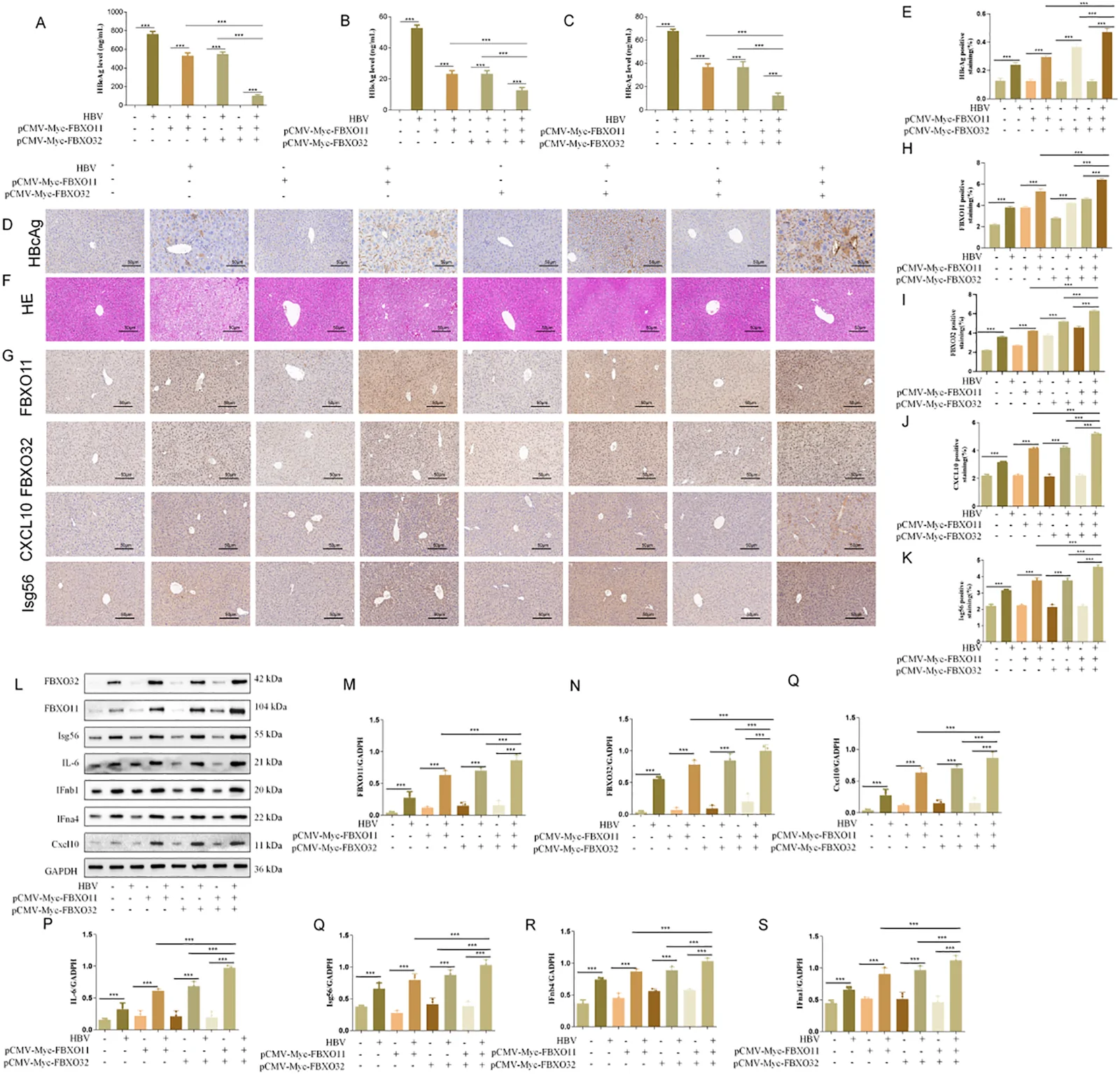
Double overexpression of FBXO11 and FBXO32 during HBV infection enhances synergistic innate immunity, leading to reduced viral antigen levels and alleviated liver pathology. **(A–C)** The level of HBsAg, HBeAg and HBcAg in mice serum at 24h post-HBV infection. **(D–E)** The level of HBcAg in liver tissues from mice at24h post-HBV infection. **(F)** HE staining of liver tissues from mice at 24 post-HBV infection. **(G–K)** Immunohistochemical staining results of FBXO11, FBXO32, and immune-related markers (Isg56, Cxcl10) in liver tissues from mice at 24h post-HBV infection. **(L–S)** The protein levels of FBXO11, FBXO32, Cxcl10, Il6, Isg56, Ifnb1, Ifna4 were measured by Western blot in mice at 24h post-HBV infection. n ≥3, *P<0.05, **P<0.001, ***P<0.0001.

Building on the above experiments, we performed overexpression experiments by overexpressing FBXO11 and/or FBXO32 *in vivo*. Upon HBV infection, serum HBsAg, HBeAg and HBcAg levels were markedly elevated; however, double overexpression of FBXO11 and FBXO32 significantly enhanced viral clearance, leading to persistently lower serum HBsAg,HBeAg and HBcAg compared with single−gene overexpression (**Figures 4A–C**). Immunohistochemical staining for HBcAg in liver tissue was consistent with the serum findings (**Figures 4D, E**). Furthermore, histopathological analysis revealed that HBV−induced inflammatory infiltration, edema, and degeneration were partially alleviated by single overexpression and substantially improved by double overexpression (**Figure 4F**). In addition, immunohistochemical staining showed that the antiviral effectors ISG56 and CXCL10, which were moderately induced by HBV alone, were further increased by single overexpression and were elevated to an even greater extent upon double overexpression (**Figures 4G–K**). Consistently, Western blot analysis confirmed that the upregulation of Ifnb1, Ifna4, ISG56, CXCL10, and IL−6 was significantly enhanced by single overexpression, and was further increased to a greater extent upon double overexpression (**Figures 4L–S**). Collectively, these overexpression experiments demonstrate that FBXO11 and FBXO32 act synergistically to potentiate antiviral immunity, suppress HBV replication, and ameliorate virus−induced liver pathology.

## 4 Discussion

F-box proteins function as substrate-recognition subunits of SCF ubiquitin ligase complexes and play critical roles in cell cycle regulation, signal transduction, and protein homeostasis (28, 29, 32). However, their functional network in antiviral innate immunity, particularly within the IFN-I signaling pathway, has only recently begun to be elucidated (30, 31). A previous study demonstrated that FBXO11 promotes K63-linked polyubiquitination of TRAF3 in a NEDD8-dependent manner, thereby facilitating the assembly of the TRAF3–TBK1–IRF3 complex (27). Building upon this foundation, the present study makes significant progress by identifying, for the first time, another F-box family member, FBXO32, which is specifically induced during the immune response to HBV infection and acts synergistically with FBXO11 to enhance host antiviral defense. This discovery not only reveals the functional diversity of F-box proteins in immune regulation but also suggests potential synergy among family members in response to pathogen invasion.

A key finding of this study is the specific upregulation of FBXO11 and FBXO32 expression upon HBV infection. This phenomenon carries important pathophysiological implications. As a hepatotropic DNA virus, HBV can be recognized by the cytosolic DNA sensor cGAS via its replication intermediates or by RLRs sensing its RNA, thereby activating the downstream MAVS/STING–TBK1–IRF3 signaling axis (11–14). Recent studies have highlighted that the cGAS-STING pathway is particularly critical for detecting HBV DNA during active replication (12, 14). Moreover, TLRs expressed on hepatocytes and immune cells also recognize distinct HBV components, with TLR3 recognizing HBV double-stranded RNA intermediates, and TLR7/9 sensing HBV single-stranded RNA and CpG DNA motifs, respectively (9, 10). Upon activation, TLR signaling converges on TRAF3, which serves as a critical adaptor orchestrating the downstream TBK1-IRF3 axis (19–21). The TLR3-TRIF pathway recruits TRAF3 to activate TBK1 and IRF3, while TLR7/9 utilize MyD88 to engage TRAF3 for type I interferon production (22, 23). The upregulation of FBXO11 and FBXO32—both components of E3 ligase complexes—may represent an adaptive host response aimed at rapidly amplifying and consolidating this initial antiviral signal. Loss-of-function experiments provide direct evidence for this hypothesis: double knockout of FBXO11 and FBXO32 more potently suppressed HBV-induced expression of IFN-β and ISGs (ISG56, CXCL10) than did knockout of either gene alone. Importantly, control experiments detecting the vector backbone sequence confirmed that the majority of HBV DNA detected at 24 hours post-infection represented progeny virus rather than residual input plasmid, further supporting the reliability of our *in vivo* model and findings.

Previous mechanistic studies have shown that FBXO11 specifically catalyzes K63-linked polyubiquitination of TRAF3, a process dependent on the NEDD8-mediated neddylation system (27, 35). K63-linked ubiquitin chains typically do not target substrates for proteasomal degradation but instead serve as molecular “scaffolds” or “activation switches” that promote protein complex assembly and signal transduction (26, 27, 36). The present study confirms that FBXO32 also participates in this precisely regulated process: in FBXO32-deficient cells, the ability of NEDD8 to promote TRAF3 ubiquitination was markedly impaired. These data strongly support the notion that FBXO11 and FBXO32 share a common NEDD8-centered regulatory module for TRAF3 ubiquitination. In this context, it is noteworthy that pharmacological inhibition of neddylation, the conjugation of NEDD8 to target proteins, has been shown to exert direct antiviral effects against HBV replication. Abounouh et al. demonstrated that blocking the NEDD8-activating enzyme (NAE) with MLN4924 reduces HBV replication *in vitro* and *in vivo*, suggesting that neddylation is a host dependency factor for HBV (32). Our findings extend this concept by showing that FBXO11 and FBXO32, two F-box proteins that depend on NEDD8 for their E3 ligase activity, synergistically promote TRAF3 ubiquitination and IFN-I signaling. Thus, while global inhibition of neddylation may suppress HBV replication through pleiotropic effects, our study identifies a specific neddylation-dependent mechanism-namely, the FBXO11/FBXO32-TRAF3 axis—that contributes to host antiviral defense. These observations collectively underscore the complex, context-dependent role of neddylation in HBV infection: neddylation is required for optimal innate immune activation via FBXO11/FBXO32, yet its excessive or dysregulated activity may also benefit viral replication, as suggested by the antiviral effect of NAE inhibitors. Further investigation into the precise spatiotemporal regulation of neddylation in hepatocytes will be necessary to reconcile these findings and to develop targeted therapeutic strategies. A plausible model is that upon HBV stimulation, FBXO11 and FBXO32 may be recruited to the same SCF complex or form a functional complex that acts cooperatively on TRAF3, enhancing the efficiency or stability of its ubiquitination. This would in turn more effectively promote TRAF3–TBK1 interaction and amplify the activation signal of the TBK1–IRF3 axis.

The *in vivo* component of this study provides compelling physiological corroboration for the above cellular and molecular mechanisms. In an HBV-infected mouse model, knockout of FBXO11 and FBXO32 resulted in synergistically exacerbated pathology. Histopathologically, double knockout significantly aggravated HBV-induced hepatic inflammatory infiltration, edema, and parenchymal damage, consistent with an impaired local antiviral state resulting from their deficiency. Virologically, the decline in serum HBsAg,HBeAg and HBcAg levels was significantly delayed in the double-knockout group compared with single-knockout groups, and immunohistochemical staining for HBcAg in liver tissue was consistent with these serum findings, directly demonstrating the importance of the synergistic action of these two proteins for effective viral suppression. In terms of immune response dynamics, the expression of FBXO11, FBXO32, and their downstream effector molecules (e.g., IFNB1, ISG56) in liver tissues exhibited highly synchronized temporal patterns. Notably, the expression of the cytokine IL6 also showed synergistic upregulation. Given the complex and multifaceted roles of IL6 in inflammation and immunity, its specific function in this synergistic antiviral response warrants further investigation (39–41). Conversely, gain-of-function experiments in which FBXO11 and FBXO32 were overexpressed further strengthened this conclusion. Double overexpression not only reduced serum HBsAg,HBeAg and HBcAg levels and ameliorated liver pathology but also enhanced the expression of ISG56, CXCL10, and type I interferon effectors, with immunohistochemical staining results in liver tissue similarly corroborating the serological findings, phenocopying the opposite effect of double knockout. These reciprocal observations establish that FBXO11 and FBXO32 are both necessary and sufficient for synergistic antiviral immunity against HBV.

In summary, moving beyond the traditional understanding of individual F-box protein functions, this study reveals for the first time the NEDD8-dependent synergistic action of FBXO11 and FBXO32 in anti-HBV innate immunity. By cooperatively enhancing K63-linked ubiquitination of TRAF3—a key event in the signaling cascade—these two proteins act as “molecular amplifiers” that synergistically elevate the activation threshold and efficiency of the TBK1–IRF3 axis, thereby endowing the host with a more robust early antiviral defense capability. This discovery not only deepens our understanding of the fine-tuning mechanisms underlying innate immune signaling but also provides a new conceptual framework for the development of innovative immunotherapeutic strategies against HBV and other viral infections. Simultaneously targeting or enhancing the function of FBXO11 and FBXO32, or modulating their common upstream node NEDD8, represents a promising therapeutic approach to synergistically suppress viral replication by potentiating host intrinsic immunity.

## Data Availability

The raw data supporting the conclusions of this article will be made available by the authors, without undue reservation.
